# Supplementary figures and images for: Genetic gradual reduction of OGT activity unveils the essential role of O-GlcNAc in the mouse embryo
Source: PLoS Genet. 2025 Jan 9;21(1):e1011507. doi: 10.1371/journal.pgen.1011507 (PMC11717234; doi:10.1371/journal.pgen.1011507)

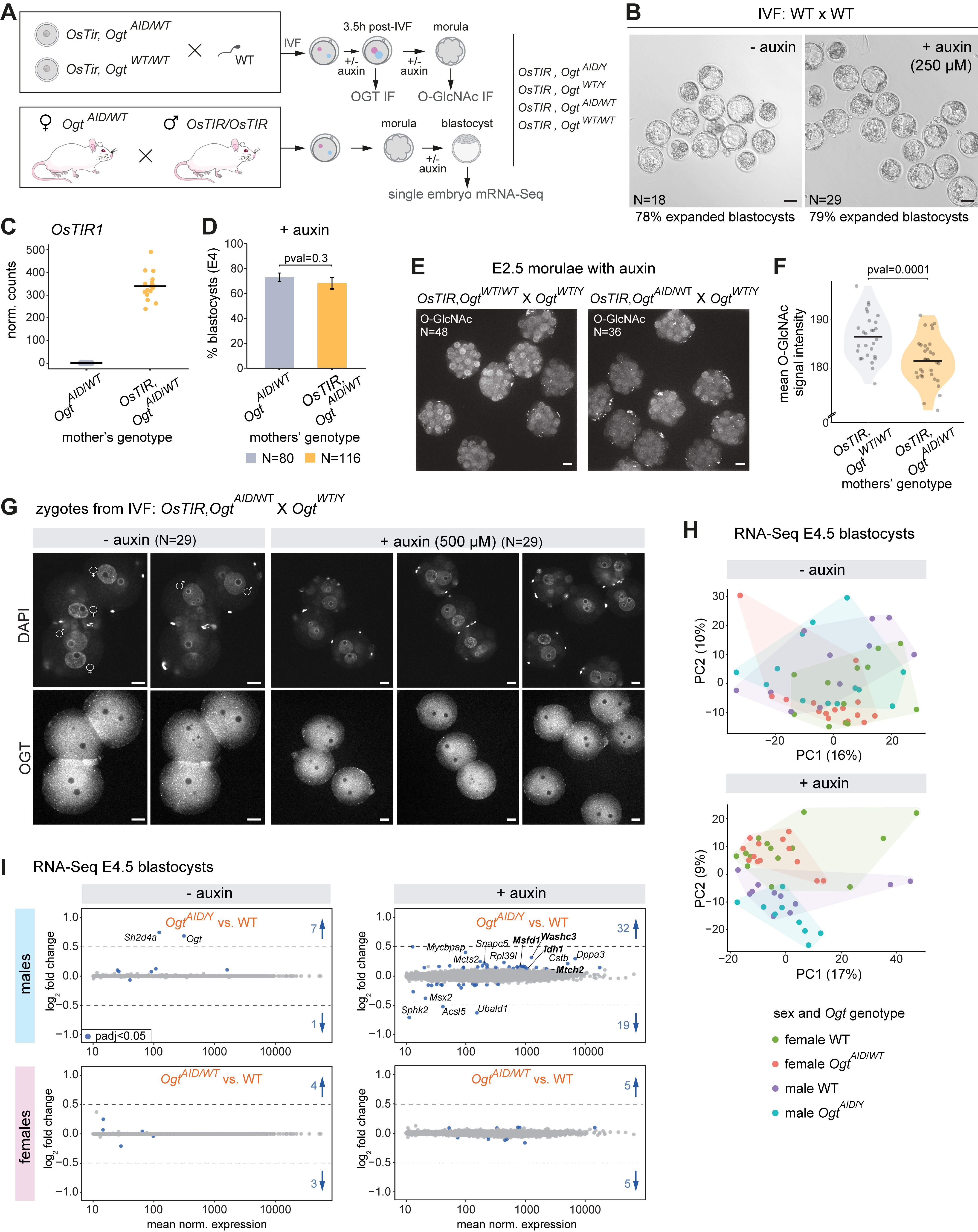

Supplement: S5 Fig — (A) Scheme of the experiments performed to test AID-OGT degradation ex vivo in preimplantation embryos. Top: IVF between WT sperm and either OgtWT/WT or OgtAID/WT OsTIR-expressing females was used to produce embryos untreated or treated with auxin from the fertilization plate, which were stained for OGT and O-GlcNAc at the zygote and morula stages, respectively. Bottom: natural mating of OgtAID/WT females with OsTIR-homozygous males was used to produce zygotes grown ex-vivo until the blastocyst stage for single embryo mRNA-Seq; 24 hours before collection, half of them were moved to a medium supplemented with 250 μM auxin. In both types of experiment, females heterozygous and males homozygous AID-Ogt as well as control Ogt WT embryos, all expressing OsTIR, are produced for analysis. (B) Test for auxin toxicity on WT embryos. Embryos were generated through IVF and cultured ex vivo in the absence or presence of auxin from the moment of fertilization to E4. Representative widefield microscopy images are shown for both conditions, total number of starting zygotes and percentage of E4 expanded blastocysts are indicated. Scale bar indicates 40 μm. (C) DESeq2-normalized gene counts of OsTIR1 for E4 blastocysts obtained from IVF of OsTIR,OgtAID (N = 19 blastocysts) or control OgtAID oocytes (not bearing the OsTIR1 gene; N = 15 blastocysts) with WT sperm. The mean for each group of embryos is drawn. (D) Percentage of E4 blastocysts obtained from the same IVF as in (C). Embryos of both groups were treated with auxin from the time of fertilization to E4. Barplot heights and error bars indicate the mean and standard deviation, respectively, of three replicate experiments of IVF followed by auxin treatment. Total number of starting 2-cell embryos for the two groups is stated in the legend. P-value is for paired two-sided Student’s t-test, assuming unequal variance. (E) Representative images of O-GlcNAc immunofluorescence staining (RL2 antibody) in morulae from OsTIR,OgtAID/WT and [file pgen.1011507.s005.tif]
