# Supplementary material for: Genetic gradual reduction of OGT activity unveils the essential role of O-GlcNAc in the mouse embryo
Source: PLoS Genet. 2025 Jan 9;21(1):e1011507. doi: 10.1371/journal.pgen.1011507 (PMC11717234; doi:10.1371/journal.pgen.1011507)
Supplement: S1 Table — (DOCX) [file pgen.1011507.s007.docx]

**Table S1: Description of the murine alleles generated.**

| **Allele name** | | **Mutation type** | **Targeted exon** | **Genomic coordinates insertion or substitution (GRCm39/mm39)** |
| --- | --- | --- | --- | --- |
| Short name | Standardized nomenclature |  |  |  |
| *Ogt^Y851A^* | FVB/NCrl-*Ogt*^em1^*^(Y851A)^*Emr | substitution Y851A | exon 19 (*Ogt-201*) | substitution: GTACTGTAACTTTAATCAGTTATATAAAATTGACCCATCT -> CTATTGCAATTTCAACCAACTGGCCAAGATCGATCCTAGC, ChrX:100719847->100719886 forward strand |
| *Ogt^T931A^* | FVB/NCrl-*Ogt*^em2^*^(T931A)^*Emr | substitution T931A | exon 20 (*Ogt-201*) | substitution: CACC -> TGCT, ChrX:100722515->100722518 forward strand |
| *Ogt^Q949N^* | FVB/NCrl-*Ogt*^em3^*^(Q949N)^*Emr | substitution Q949N | exon 19 (*Ogt-201*) | substitution: GTACTGTAACTTTAATCAGTTATATAAAATTGACCCATCT -> CTATTGCAATTTCAACAACCTGTACAAGATCGATCCTAGC, ChrX:100719847->100719886 forward strand |
| *Ogt^H568A^* | FVB/NCrl-*Ogt*^em4^*^(H568A)^*Emr | substitution H568A | exon 13 (*Ogt-201*) | substitution: GAATCAC -> GAATGCT, ChrX:100713458->100713464 forward strand |
| *Ogt^NLS-^* | FVB/NCrl-*Ogt*^em5^*^(DFPmut)^*Emr | substitution DFP(461-463) -> AAA | exon 11 (*Ogt-201*) | substitution: GACTTTCCT -> GCTGCTGCA, ChrX:100711182->100711190 forward strand |
| *Ogt^NterAID-MYC-FLAG^* | FVB/NCrl-*Ogt*^em6^*^(AID)^*Emr | insertion [N-ter AID-MYC-FLAG] | exon 1 (*Ogt-201*) | insertion: ChrX:100683892 forward strand CCTAAAGACCCTGCAAAACCTCCCGCAAAGGCACAGGTGGTTGGATGGCCCCCAGTGAGATCCTATCGAAAGAACGTAATGGTTTCTTGTCAAAAGAGTAGTGGTGGACCTGAGGCCGCAGCCTTCGTGAAGGAACAAAAACTTATCAGCGAGGAAGACCTCGAGCAGAAGCTGATCAGCGAGGAAGACCTGGATTATAAAGACGACGATGATAAA |
| ROSA26*^OsTIR^* | FVB;B6J;129-Gt(ROSA)26Sor^tm1^*^(OsTIR)^*Emr | Insertion [OsTIR-Myc-HA] | ROSA26 locus | Insertion: Chr6:113,076,033 (mm10): ATCTGTAGGGCGCAGTAGTCCAGGGTTTCCTTGATGATGTCATACTTATCCTGTCCCTTTTTTTTCCACAGCTCGCGGTTGAGGACAAACTCTTCGCGGTCTTTGTGCACTTAAGATAACTTCGTATAGCATACATTATACGAAGTTATCCAGTGGGGATCGACGGTATCGATAAGCTTCCACCATGACATACTTTCCTGAAGAGGTCGTCGAACACATTTTTAGCTTCCTGCCTGCACAGAGAGATAGAAACACAGTGAGCCTGGTCTGCAAAGTGTGGTACGAGATCGAACGCCTGAGCCGGAGAGGAGTGTTCGTCGGCAACTGCTATGCTGTGAGAGCAGGCAGGGTCGCCGCTAGGTTTCCAAATGTGCGCGCACTGACCGTCAAGGGGAAACCCCACTTCGCCGACTTTAACCTGGTGCCCCCTGATTGGGGAGGATACGCCGGCCCTTGGATCGAGGCAGCCGCTCGCGGCTGTCATGGACTGGAGGAACTGCGCATGAAGCGAATGGTGGTCTCTGACGAAAGTCTGGAGCTGCTGGCTCGGAGCTTCCCTAGGTTTCGCGCACTGGTGCTGATTTCTTGCGAAGGCTTCAGCACCGATGGACTGGCAGCCGTGGCCTCCCACTGTAAGCTGCTGCGGGAGCTGGACCTCCAGGAGAATGAAGTGGAGGATAGAGGCCCCAGATGGCTGTCTTGCTTCCCAGACTCATGTACCAGCCTGGTGTCCCTGAACTTTGCCTGCATCAAAGGCGAAGTGAATGCTGGGTCCCTGGAGCGGCTGGTCTCAAGAAGCCCCAACCTGAGGTCTCTGCGGCTGAACCGGAGCGTGAGCGTGGACACTCTGGCTAAGATTCTGCTGAGAACCCCTAACCTGGAGGATCTGGGAACCGGCAATCTGACAGACGATTTCCAGACAGAATCCTACTTTAAACTGACTTCTGCCCTGGAGAAGTGTAAAATGCTGAGGAGTCTGTCAGGATTCTGGGATGCTTCACCCGTGTGCCTGAGCTTTATCTACCCTCTGTGTGCACAGCTGACAGGCCTGAACCTGAGCTATGCACCAACCCTGGACGCCAGTGATCTGACAAAGATGATCTCACGCTGCGTGAAACTCCAGCGACTGTGGGTGCTGGACTGTATTTCCGATAAGGGGCTCCAGGTGGTCGCCAGCTCCTGCAAGGACCTCCAGGAGCTGAGAGTGTTCCCATCTGATTTTTACGTGGCCGGATATAGTGCTGTCACTGAGGAAGGCCTGGTGGCAGTCTCACTGGGATGCCCAAAGCTGAACAGCCTGCTGTATTTCTGTCATCAGATGACTAATGCTGCACTGGTGACCGTCGCCAAGAACTGCCCTAATTTCACCCGATTTCGGCTGTGTATTCTGGAACCAGGCAAACCCGACGTGGTCACATCCCAGCCACTGGATGAAGGGTTTGGAGCTATCGTGAGAGAGTGCAAGGGACTCCAGAGGCTGAGCATTTCCGGCCTGCTGACAGACAAAGTGTTCATGTACATCGGCAAGTATGCTAAGCAGCTGGAGATGCTGAGCATTGCATTTGCCGGAGACTCCGATAAGGGCATGATGCACGTGATGAACGGGTGTAAGAATCTGCGAAAACTGGAAATCCGGGACAGCCCTTTCGGGGATGCCGCTCTGCTGGGAAACTTTGCCAGATACGAGACAATGAGGAGCCTGTGGATGTCTAGTTGCAATGTGACTCTGAAGGGCTGTCAGGTCCTGGCTAGTAAAATGCCTATGCTGAACGTGGAAGTCATTAATGAGCGGGACGGGTCTAACGAAATGGAGGAAAATCATGGCGACCTGCCAAAGGTGGAGAAACTGTATGTGTATCGGACCACCGCAGGGGCAAGAGATGATGCTCCCAACTTTGTGAAGATTCTGGAGGAGCAGAAGCTGATCTCAGAGGAGGACCTGTACCCATACGATGTTCCAGATTACGCTGTCGACTAGCATATGTACGAAGTTATAAGCTGGAAGTTCCTATTCTCTAGAAAGTATAGGAACTTCAAGCTTAGGTGGCACTTTTCGGGCTACCGGGTAGGGGAGGCGCTTTTCCCAAGGCAGTCTGGAGCATGCGCTTTAGCAGCCCCGCTGGGCACTTGGCGCTACACAAGTGGCCTCTGGCCTCGCACACATTCCACATCCACCGGTAGGCGCCAACCGGCTCCGTTCTTTGGTGGCCCCTTCGCGCCACCTTCTACTCCTCCCCTAGTCAGGAAGTTCCCCCCCGCCCCGCAGCTCGCGTCGTGCAGGACGTGACAAATGGAAGTAGCACGTCTCACTAGTCTCGTGCAGATGGACAGCACCGCTGAGCAATGGAAGCGGGTAGGCCTTTGGGGCAGCGGCCAATAGCAGCTTTGCTCCTTCGCTTTCTGGGCTCAGAGGCTGGGAAGGGGTGGGTCCGGGGGCGGGCTCAGGGGCGGGCTCAGGGGCGGGGCGGGCGCCCGAAGGTCCTCCGGAGGCCCGGCATTCTGCACGCTTCAAAAGCGCACGTCTGCCGCGCTGTTCTCCTCTTCCTCATCTCCGGGCCTTTCGACCTGCAGCAGCACGTGTTGACAATTAATCATCGGCATAGTATATCGGCATAGTATAATACGACAAGGTGAGGAACTAAACCATGGGATCGGCCATTGAACAAGATGGATTGCACGCAGGTTCTCCGGCCGCTTGGGTGGAGAGGCTATTCGGCTATGACTGGGCACAACAGACAATCGGCTGCTCTGATGCCGCCGTGTTCCGGCTGTCAGCGCAGGGGCGCCCGGTTCTTTTTGTCAAGACCGACCTGTCCGGTGCCCTGAATGAACTGCAGGACGAGGCAGCGCGGCTATCGTGGCTGGCCACGACGGGCGTTCCTTGCGCAGCTGTGCTCGACGTTGTCACTGAAGCGGGAAGGGACTGGCTGCTATTGGGCGAAGTGCCGGGGCAGGATCTCCTGTCATCTCACCTTGCTCCTGCCGAGAAAGTATCCATCATGGCTGATGCAATGCGGCGGCTGCATACGCTTGATCCGGCTACCTGCCCATTCGACCACCAAGCGAAACATCGCATCGAGCGAGCACGTACTCGGATGGAAGCCGGTCTTGTCGATCAGGATGATCTGGACGAAGAGCATCAGGGGCTCGCGCCAGCCGAACTGTTCGCCAGGCTCAAGGCGAGCATGCCCGACGGCGAGGATCTCGTCGTGACCCATGGCGATGCCTGCTTGCCGAATATCATGGTGGAAAATGGCCGCTTTTCTGGATTCATCGACTGTGGCCGGCTGGGTGTGGCGGACCGCTATCAGGACATAGCGTTGGCTACCCGTGATATTGCTGAAGAGCTTGGCGGCGAATGGGCTGACCGCTTCCTCGTGCTTTACGGTATCGCCGCTCCCGATTCGCAGCGCATCGCCTTCTATCGCCTTCTTGACGAGTTCTTCTGAGCGGGACTCTGGGGTTCGAAATGACCGACCAAGCGACGCCCAACCTGCCATCACGAGATTTCGATTCCACCGCCGCCTTCTATGAAAGGTTGGGCTTCGGAATCGTTTTCCGGGACGCCGGCTGGATGATCCTCCAGCGCGGGGATCTCATGCTGGAGTTCTTCGCCCACCCTAGGGGGAGGCTAACTGAAACACGGAAGGAGACAATACCGGAAGGAACCCGCGCTATGACGGCAATAAAAAGACAGAATAAAACGCACGGTGTTGGGTCGTTTGTTCATAAACGCGGGGTTCGGTCCCAGGGCTGGCACTCTGTCGATACCCCACCGAGACCCCATTGGGGCCAATACGCCCGCGTTTCTTCCTTTTCCCCACCCCACCCCCCAAGTTCGGGTGAAGGCCCAGGGCTCGCAGCCAACGTCGGGGCGGCAGGCCCTGCCAGGATCCGAAGTTCCTATTCTCTAGAAAGTATAGGAACTTCCTCGAGTTTAAACTAAGCTGATCAGCCTCGACTGTGCCTTCTAGTTGCCAGCCATCTGTTGTTTGCCCCTCCCCCGTGCCTTCCTTGACCCTGGAAGGTGCCACTCCCACTGTCCTTTCCTAATAAAATGAGGAAATTGCATCGCATTGTCTGAGTAGGTGTCATTCTATTCTGGGGGGTGGGGTGGGGCAGGACAGCAAGGGGGAGGATTGGGAAGACAATAGCAGGCATGCTGGGGATGCGGTGGGCTTATGGCTTCTGAGGCGGAAAGAACCAGCTGGGGCTCGAT |
