# Supplementary material for: Genetic gradual reduction of OGT activity unveils the essential role of O-GlcNAc in the mouse embryo
Source: PLoS Genet. 2025 Jan 9;21(1):e1011507. doi: 10.1371/journal.pgen.1011507 (PMC11717234; doi:10.1371/journal.pgen.1011507)
Supplement: S2 Table — (DOCX) [file pgen.1011507.s008.docx]

**Table S2. List of primers for genotyping the murine alleles.**

| **Allele target** | **Primer direction** | **Sequence (5’-3’)** |
| --- | --- | --- |
| *Ogt^NterAID-MYC-FLAG^* | forward | AGTAGTGGCGGCAGTAGAAG |
|  | reverse | TAATGGGGATGGTCAGAGGG |
| *OsTIR* insert (to genotype for the presence of the insert at least on one allele) | forward | AGAGATAGAAACACAGTGAGCC |
|  | reverse | TCGCAAGAAATCAGCACCAG |
| *OsTIR* flanking sequence (to genotype for homozygosity, if used together with primers in row above) | forward | AGTCGCTCTGAGTTGTTATCAG |
|  | reverse | AGGTTAGCCTTTAAGCCTGC |
| *Ogt^Q949N^* | forward | CTTGACTCAAAACCAGGGCC |
| *Ogt^Q949N^* | reverse | ATGGGGAAGGGAGATTCAGC |
| *Ogt^Y851A^* | forward | CTTGACTCAAAACCAGGGCC |
|  | reverse | ATGGGGAAGGGAGATTCAGC |
| *Ogt^WT^* allele and *Ogt^T931A^* allele at the region containing T931* | reverse | CATGTGGTCAGGTTTGTTGC |
|  | forward | GCGTTTTCCAGCAGTAGGA |
| only *Ogt^T931A^* allele* | reverse | GAACATCCATCCCTGTAGCA |
|  | forward | GCGTTTTCCAGCAGTAGGA |

* both pairs of primers were used for genotyping of the *Ogt^T931A^* mouse line: a positive signal with the first pair is necessary to interpret a negative signal with the second pair as a true wild type.
