# Supplementary material for: Genetic gradual reduction of OGT activity unveils the essential role of O-GlcNAc in the mouse embryo
Source: PLoS Genet. 2025 Jan 9;21(1):e1011507. doi: 10.1371/journal.pgen.1011507 (PMC11717234; doi:10.1371/journal.pgen.1011507)
Supplement: S3 Table — (DOCX) [file pgen.1011507.s009.docx]

**Table S3. List of primers for sexing and genotyping the MEFs.**

| **Gene target** | **Primer direction** | **Sequence (5’-3’)** |
| --- | --- | --- |
| *Ogt^NterAID-MYC-FLAG^* | forward | AGTAGTGGCGGCAGTAGAAG |
|  | reverse | TAATGGGGATGGTCAGAGGG |
| *OsTIR* | forward | AGAGATAGAAACACAGTGAGCC |
|  | reverse | TCGCAAGAAATCAGCACCAG |
| *Sly-Xlr* (McFarlane et al., 2013) | forward | GATGATTTGAGTGGAAATGTGAGGTA |
|  | reverse | CTTATGTTTATAGGCATGCACCATGTA |
