# Supplementary material for: Genetic gradual reduction of OGT activity unveils the essential role of O-GlcNAc in the mouse embryo
Source: PLoS Genet. 2025 Jan 9;21(1):e1011507. doi: 10.1371/journal.pgen.1011507 (PMC11717234; doi:10.1371/journal.pgen.1011507)
Supplement: S4 Table — (DOCX) [file pgen.1011507.s010.docx]

**Table S4. List of primers for sexing and genotyping Ogt*^T931A^* blastocysts.**

| **Gene target** | **Primer direction** | **Sequence (5’-3’)** |
| --- | --- | --- |
| Xist (cDNA) | forward | TCTATCTTGTGGGTCCTGGAG |
|  | reverse | CTCCTCTAAATCCAGGCAATCC |
| Ddx3y (cDNA) | forward | TGGAGGAGGAAATACAGAGAGC |
|  | reverse | GGAGGACAATTATTTCCAGTTGC |
| Eif2s3y (cDNA) | forward | TGGCTGTGAAGTTGATGACC |
|  | reverse | CCTTCTGTACGTACACCTAGG |
| *Ogt^+^* allele and *Ogt^T931A^* allele* | reverse | CATGTGGTCAGGTTTGTTGC |
|  | forward | GCGTTTTCCAGCAGTAGGA |
| only *Ogt^T931A^* allele* | reverse | GAACATCCATCCCTGTAGCA |
|  | forward | GCGTTTTCCAGCAGTAGGA |

* both pairs of primers were used for genotyping of the *Ogt^T931A^* blastocysts: a positive signal with the first pair is necessary to interpret a negative signal with the second pair as a true wild type.
