# Supplementary material for: Genetic gradual reduction of OGT activity unveils the essential role of O-GlcNAc in the mouse embryo
Source: PLoS Genet. 2025 Jan 9;21(1):e1011507. doi: 10.1371/journal.pgen.1011507 (PMC11717234; doi:10.1371/journal.pgen.1011507)
Supplement: S5 Table — (DOCX) [file pgen.1011507.s011.docx]

**Table S5. Details on the generation and filtering steps of the single embryo Smart-Seq datasets.**

| **Mouse line** | **Type of sequencing** | **# pooled embryos in sequencing run** | **Average # of reads per embryo** | **# pooled embryos in sequencing run, per condition** | | **# embryos discarded for < 10^6^ reads** | **# embryos discarded for high rDNA/mt gene counts** | **# embryos discarded for uncertain genotype** | **# embryos discarded for visible batch effect** | **# embryos for DE analysis** |
| --- | --- | --- | --- | --- | --- | --- | --- | --- | --- | --- |
| *Ogt^NterAID-MYC-FLAG^* | 75 bp SE | 119 | 4.5* 10^6^ | AUX | 60 | 2 | 0 | 1 | 10 | 47 |
|  |  |  |  | UNT | 59 | 0 | 0 | 0 | 7 | 52 |
| *Ogt^T931A^* | 40 bp PE | 71 | 8.6* 10^6^ | WT mothers | 37 | 5 | 0 | 1 | n.a. | 31 |
|  |  |  |  | *Ogt^T931A/+^* mothers | 34 | 4 | 0 | 0 | n.a. | 30 |

### 
