# Supplementary material for: Genetic gradual reduction of OGT activity unveils the essential role of O-GlcNAc in the mouse embryo
Source: PLoS Genet. 2025 Jan 9;21(1):e1011507. doi: 10.1371/journal.pgen.1011507 (PMC11717234; doi:10.1371/journal.pgen.1011507)
Supplement: S1 Text — (DOCX) [file pgen.1011507.s012.docx]

### **Supplementary text**

### The AID-OGT degron system is inefficient in *ex vivo* grown preimplantation embryos

We obtained zygotes with all relevant genotypes (i.e. females *OsTIR*,*Ogt^WT/WT^* and *OsTIR*,*Ogt^AID/WT^*; males *OsTIR*,*Ogt^WT/^*^Y^ and *OsTIR*,*Ogt^AID/^*^Y^) by IVF of oocytes derived from *OsTIR*-homozygous,*Ogt^AID/WT^* females or control *OsTIR*-homozygous,*Ogt^WT/WT^* littermates (using WT sperm). Half of the embryos were cultured in presence of auxin from fertilization to the morula stage (embryonic day 2.5; Fig. S5A, top row). We had previously determined that the experimental concentration of auxin was not toxic to the embryos (Fig. S5B) and that the *OsTIR* transgene was expressed in blastocysts from *OsTIR*,*Ogt^AID/WT^* oocytes (Fig. S5C). Moreover, the rate of formation of expanded blastocysts from zygotes grown in auxin was not significantly different between *OsTIR*,*Ogt^AID/WT^* and control oocytes (Fig. S5D), hence all four possible genotypes should be equally represented in the population of morulae. OGT depletion is predicted to cause a reduction of global levels of O-GlcNAc in *OsTIR*,*Ogt^AID/^*^Y^ males (~25% of the embryos), which do not bear any WT *Ogt* gene. We quantified the O-GlcNAc immunofluorescence (IF) signal in single morulae from *OsTIR*,*Ogt^AID/WT^* mothers after auxin treatment and found that the signal was only slightly diminished (Fig. S5E,F). Using the same breeding scheme, we also stained OGT in zygotes (3.5 hours post-IVF) cultured in auxin from fertilization. We chose this stage because the OGT signal is enriched in the paternal pronucleus (Fig. S5G, no auxin), enabling an easier qualitative assessment of OGT reduction. None of the auxin-treated zygotes displayed a clearly diminished OGT signal, even with a higher concentration of auxin (Fig. S5G, N = 29).

We could not exclude a low level of auxin-induced OGT degradation below immunofluorescence sensitivity. Thus, we investigated whether a molecular difference emerged in auxin-treated *OsTIR*,*Ogt^AID/^*^Y^ embryos using transcriptomics. We collected zygotes recovered from crosses of *Ogt^AID/WT^* females with *OsTIR*-homozygous males, cultured them *ex vivo* for 72 hours, then treated half of the morulae with auxin for 24 hours and collected the blastocysts for single embryo mRNA-Seq (Fig. S5A, bottom row). Principal component analysis (PCA) showed a partial separation between the *Ogt^WT^* and *Ogt^AID^* genotypes only for males and only in the presence of auxin (Fig. S5H). Accordingly, gene expression changes were more pronounced for hemizygous males treated with auxin (Fig. S5I). The male-specific gene expression change induced by auxin treatment provides an indirect indication that the degron system is activated and partially impacts OGT’s function. It is noteworthy that, among the 32 upregulated DEGs, 4 were also significantly upregulated at a higher degree in *Ogt^T931del/^*^Y^ blastocysts: *Washc3,* which promotes actin polymerization at the surface of endosomes, the lysosomal transporter *Mfsd1*, mitochondrial insertase *Mtch2* and isocitrate dehydrogenase 1 (*Idh1*) (in bold in Fig. S5I). This further supports auxin-induced depletion of OGT, although to a mild extent. However, the resulting transcriptional change is of low magnitude and high variance, which results in a low number of DEGs (Fig. S5I) and prevents drawing conclusions about OGT’s function in the blastocyst using this system.

In conclusion, indirect transcriptomic evidence shows that in preimplantation embryos the AID degron induces OGT depletion, but the O-GlcNAc perturbation is suboptimal in this *ex vivo* model. It is worth mentioning that recently optimized versions of the AID system might achieve a better result in preimplantation embryos [[1]](https://app.readcube.com/library/e7cecd59-2395-47d5-8fc1-01eaee9ac961/all?uuid=5923763959036731&item_ids=e7cecd59-2395-47d5-8fc1-01eaee9ac961:411719da-8549-4c2b-b0b3-7a582e4f006f).

**Supplementary text reference**

[1. Yesbolatova A, Saito Y, Kitamoto N, Makino-Itou H, Ajima R, Nakano R, et al. The auxin-inducible degron 2 technology provides sharp degradation control in yeast, mammalian cells, and mice. Nature Communications. 2020;11: 1–13. doi:10.1038/s41467-020-19532-z](https://app.readcube.com/library/?style=PLOS%20Genetics+%7B%22language%22:%22en-US%22%7D)
