# Supplementary material for: Genetic gradual reduction of OGT activity unveils the essential role of O-GlcNAc in the mouse embryo
Source: PLoS Genet. 2025 Jan 9;21(1):e1011507. doi: 10.1371/journal.pgen.1011507 (PMC11717234; doi:10.1371/journal.pgen.1011507)
Supplement: Attachment — Submitted filename: 146516_1_rebuttal_3105885_shsgsp.pdf [file pgen.1011507.s013.pdf]

Full Revision

Review COMMONS

Manuscript number: RC-2024-02480R
Corresponding author(s): Matthieu, Boulard

1. General Statements

We would first like to thank the reviewers for their careful reading and thoughtful feedback.

We were pleased that reviewer 1 concludes their statement with "*All in all, this is an interesting study that track functions of OGT in early embryonic development. The studies are well-controlled and rigorous*", that reviewer 2 states "*The study represents a substantial advance in our understanding of OGT and O-GlcNAcylation in mammalian development. The creation of novel murine models and inducible systems is an important contribution, providing powerful tools for future research in this field.";* a view that is shared by reviewer 3 *"The biological reagents produced and characterized will be of general interest to the field."* and reviewer 4 "*The quality of the manuscript is good because the question to be solved was appropriately set, the approach was well designed, and their findings were interesting".*

We have substantially revised the manuscript and included additional experimental evidence on O-GlcNAc and OGT/OGA protein levels in the placenta of embryos bearing the OGT-Y851A hypomorphic mutation.

Overall, we believe our improved manuscript provides compelling evidence that the glycosyltransferase activity of OGT, and thus the O-GlcNAc modification itself, plays a sexually dimorphic function in placental development and the developmental repression of retrotransposons in the developing embryo.

We have addressed each of the reviewers' comments below. The original comments (**C**) are in italic, our responses (**R**) in Roman font.

2. Point-by-point description of the revisions

Reviewer #1

Evidence, reproducibility and clarity

C1: *Formichetti at el. developed mice with OGT catalytic dead mutations and then studied their function during early embryogenesis. Not surprisingly, dramatic reduction in OGT activity failed to produce embryos; however, mild reduction in OGT did produce animals. The authors then use the T931 animals that have a mild reduction in activity to further characterize the function in the early embryo. Not surprisingly, male mice showed changes in gene expression, implantation sub-lethality, and an uptick in loss of retrotransposon silencing. The authors also show that an even milder reduction in OGT activity (Y851A) effects male placenta function and chromatin remodeling. Finally, the authors make a less stable OGT transgene within the mouse and again found embryogenesis issues in the males and alterations in numerous gene families including mTOR signaling and p53 function. All in all, this*

is an interesting study that track functions of OGT in early embryonic development. The studies are well-controlled and rigorous.

R1: We thank the reviewer for their clear understanding and their appreciation of the rigor and impact of this work.

Significance

C2: *This is a good study and novel. Not only is it of interest to reproductive biologist, but it echos themes found in O-GlcNAc biology.*

R1: We are pleased that the reviewer underlined the novelty of the study and its impact across fields.

Reviewer #2

Evidence, reproducibility and clarity

Comments to authors

C3: *To investigate the function of OGT at specific developmental stages, the authors perturbed OGT's function in vivo by creating a murine allelic series featuring four single amino acid substitutions that variably reduced OGT's catalytic activity. The goal was to identify the direct effect of O-GlcNAcylation, using a sophisticated collection of genetic mutants to evaluate in vivo the role of this modification at early stages of development. Overall, the severity of embryonic lethality correlated with the extent of catalytic impairment of OGT, demonstrating that the O-GlcNAc modification is essential for early development.*
The study represents a substantial advance in our understanding of OGT and O-GlcNAcylation in mammalian development. The creation of novel murine models and inducible systems is an important contribution, providing powerful tools for future research in this field. The insights into the role of OGT's catalytic activity and its involvement in epigenetic regulation during embryonic development are noteworthy, opening new avenues for research.

R3: We thank the reviewer for their insightful comments. We are grateful for the supporting statements. Please find below detailed response to all your comments.

However, there are a few considerations and concerns:
Major:

C4: *1. An assumption of the study is that different mutations cause different levels of O-GlcNAcylation rather than alterations in substrate specificity. It might be important to test, at least in cultured cells, that the different mutations do not change the preference of OGT to modify certain proteins rather than others, which can provide alternative explanations for their findings.*

R4: Thanks for asking this question, it helped us to better explain the rationale behind the choice of the *Ogt* amino-acid substitutions.

This is a critical point that we carefully considered in the design of the single amino-acid substitutions. Two lines of evidence support that the precise mutations created impact the catalytic rate without modifying the substrate specificity:

First, as explained in the text, the choice of the single amino-acid substitutions was driven by previous structural and enzymology knowledge. The impact of the four point mutations selected on OGT protein stability and on the Michaelis-Menten kinetic values had previously been determined experimentally (Fig. 1A legend and Martinez-Fleites, C. *et al. Nature Structure Molecular Biology* 2008; https://doi.org/10.1038/nsmb.1443).

There is a second important rationale that we added in the revised manuscript: the four point mutations selected are all located in the catalytic domain (specifically, H568A in the N-Cat domain and Y851A, T931A and Q849A in the C-Cat domain), while the substrate recognition is operated via two other domains namely the intervening domain (Int-D) https://doi.org/10.1038/s41589-023-01422-2) and the tetratricopeptide Repeat (TPR) superhelix (10.1021/jacs.7b13546; https://doi.org/10.1073/pnas.2303690120). Therefore, for both these reasons, it is extremely unlikely that these mutations could influence the substrate specificity.

C5.1: 2. *In Fig 1D and 1H, the thresholds to define a gene or TE as differentially expressed are not strong. According to the figure legends, "any" change in terms of log2Fc was considered as DE and colored. I think the figures should illustrate better that the changes are subtle, by for example adding a dotted line (at least) in the value 0.5 of the y-axis. These subtle transcriptional changes should be reflected better in certain paragraphs where the expression of TEs are presented/and discussed as a hallmark of the absence of O-GlcNAcylation in the OGT-mutants. The same happens with Suppl Fig 3C (changes are very minor). {. Applying a stronger threshold, among the upregulated genes, only Xist will be significantly overexpressed. If a gentle threshold needs to be applied to this data, authors should at least justify the reasons behind doing so. Same for Fig2D.*

R5.1: The reviewer means Figure 2D for MA plot of gene expression and Figure 2H for retrotransposons expression. These figures now include a dash line to indicate Log2FC = 0.5 (as all MA plots).

The text is explicit on the subtle changes in transcription, it reads "*with 2/3 of the genes downregulated and 90% of the significant changes below 1 log_2FC*"; "*most of the $Ogt^{T931del/Y}$ embryos showed a low magnitude upregulation of retrotransposons*".

The revised text states "*Notably, most of the OgtT931$^{del/Y}$ embryos showed a low magnitude (log2FC < 1) upregulation of retrotransposons*".

We expand on this topic in the next response (R5.2) noting that changes in gene expression upon O-GlcNAc perturbation in different systems were previously characterized as subtle and widespread. We

suggest that this phenotype may arise from the scarcely understood pleiotropic function of O-GlcNAc in fine-tuning gene expression; this phenotype could have a biological significance.

C5.2: *If a gentle threshold needs to be applied to this data, authors should at least justify the reasons behind doing so. Same for Fig2D.*

R5.2: Previous studies in different systems reported that O-GlcNAc perturbation causes a widespread change in gene expression of low magnitude (https://doi.org/10.1101/2024.01.22.576677, https://www.pnas.org/doi/10.1073/pnas.2218332120). We use the same thresholds as a recent functional *Ogt* study in ES cells to call differentially expressed genes, specifically: $p<0.05$ (Wald test), any FC (Li et al. PNAS 2023, https://www.pnas.org/doi/10.1073/pnas.2218332120). The p value threshold is standard; the absence of FC threshold is dictated by the insufficient knowledge of the significance of the low magnitude changes observed across many transcripts.

C6: *3. In Figure 2B, the T931del allele was recovered in the blastocyst population with a very high frequency, even higher than the male WT group (T931del: 10; WT: 3). This observation suggests that the T931del allele did not significantly affect blastocyst survival. Further clarification or additional experiments might be necessary to understand the implications of this finding on early developmental stages.*

R6: This is only a hint as the numbers of blastocysts recovered were too small to perform statistics on Mendelian distribution. Thus, more experiments are needed to perform these statistical tests. These experiments are onerous because the low frequency of germline transmission is incompatible with maintaining this mutation by breeding heterozygous animals. Because of this, a new mouse line needs to be created by CRISPR-HDR targeting in the zygote in order to compute statistics on Mandelian ratios. Importantly, this question - does T931del affect blastocyst survival? - is peripheral, and the results of these experiments would not affect our conclusions in any way.

C7: *4. Similarly, in Figure 2G, there is an apparent higher expression of TE expression in the T931A/Y embryos group than in the T931del/Y group, which combined with the higher frequency of blastocyst generated in this latest group it may indicate a deeper molecular consequence after the deletion of the T931. A comparison of the transcriptome between these two cell lines help to address this possibility. Also, the authors should compare the O-GlcNAc levels of WT, T931A, and T931del mutant blastocysts by immunostaining, similar to what was done in Figure S5F.*

R7: We agree that a direct comparison between the two mutations of the T931 residue would be interesting; however, this comment is very difficult to address experimentally for the reasons outlined below:

Firstly, it is not possible to perform a statistical comparison of the transcriptome *T931A/Y VS. T931del/Y* with the data generated because the number of hemizygous *T931A/Y* (n=2) is too small.

Hence, it cannot be ruled out that the seemingly milder retrotransposon reactivation in one of the *T931A/Y* embryos could have occurred by chance.

Secondly, considering the low magnitude effect on gene expression changes upon O-GlcNAc genetic perturbation, to statistically assess the penetrance of the molecular phenotype and perform the differential expression analysis, numerous (>>3) hemizygous blastocysts of each genotype would be needed. Because females heterozygous for the *T931* mutations transmit the mutant allele at very low frequency, these experiments require numerous *de novo* CRISPR injection sessions.

Thirdly, for the immunostaining of O-GlcNAc to be semi-quantitative, a large number of hemizygous blastocysts for each genotype would be required (note that in Figure S5F, 29 morulae per condition were imaged), thus requiring numerous CRISPR injection experiments as discussed above. Moreover, O-GlcNAc changes could be subtler than what expected based on the strong reduction of OGT activity, since as a compensatory mechanism *Ogt* expression is upregulated in the *Ogt*$^{T931A/del}$ blastocysts (Fig. S2D), making a quantification even more challenging despite a high number of stained embryos.

In sum, these *in vivo* experiments are difficult and require sacrificing many animals (about 20 females per CRISPR injection experiment). Because the results would bring refinement to the study but would not change our conclusions, we suggest that the cost/benefit is too high.

C8: *5. In Boulard et al. 2019 O-GlcNAcylation was shown to be sufficient to modulate expression of DNA methylation-dependent TEs. It would be interesting to know (or at least discuss) if the changes in TE expression observed in OGT-mutant embryos in this study involve changes in DNA methylation. Ideally, some DNA methylation measurement optimized for low input numbers of cells would be useful.*

R8: Thank you for making the link with our previous study. In the PNAS paper, we report that targeted removal of O-GlcNAc at proteins bound to specific TEs (e.g. IAPez) causes their full-blown reactivation without detectable changes in DNA methylation, thus suggesting a role of the O-GlcNAc modification for the silencing of methylated TEs downstream or independent of DNA methylation. We agree that it would be informative to quantify DNA methylation in the *T931*-mutant blastocysts to test if the *in vitro* result is the same *in vivo*, but this would require performing onerous microinjection sessions as explained above.

C9: *6. The data related with the OGT-degron system in MEs seem disconnected with the rest of the manuscript. While the developmental models (blastocyst, etc) elegantly assess the contribution of O-GlcNAcylation to the control of cell survival and gene expression through the use of different OGT mutants, the degron system is a system of graded depletion that unfortunately was only possible to be used in MEFs (instead of embryos). Thus, the results obtained with the degron system in MEFs are difficult to intersect with the data from the use of OGT-mutants in embryos. Even though there are obvious interesting questions that one may want to know about this OGT degron MEF system, none of them would demonstrate a direct role for O-GlcNAcylation in cellular function, the major point addressed in the developmental system. Using the degron system in embryonic stem cells might have*

provided a more parallel comparison. The authors should discuss this point in more detail and either use ESC instead of MEFs or provide a stronger justification for the use of MEFs over ESC.

R9: We thank the reviewer for their clear understanding of the system. The choice of primary MEF as an *in vitro* model was imposed by technical limitations we encountered during the study. We fully agree that ES cells is the model of choice for preimplantation embryos; thus we initially derived ES cells and obtained only one male clone bearing the AID degron system. Upon auxin addition to the culture media, OGT's level remained unchanged in ES cells. Thus, the ES cells model was not usable. To test the AID degron in a different cell type, we then derived MEFs and showed its effectiveness (Figures 4C and S4C-E), which also allowed to collect functional data on OGT's cellular function (Figures 4D-F). We took the comment on board and clarified the rationale of studying MEFs in the revised manuscript. We agree that it remains to be verified that the OGT-dependent pathways uncovered in MEFs are relevant in the preimplantation embryo. Despite this caveat, we feel the mouse model for endogenous OGT-degron, as well as the negative results *in vivo* and conclusions in MEFs should be shared with the community, which could take advantage of our results to refine the system.

Minor:

C10: *7. In Fig 2C the color and shape codes are confusing to understand - there are some colors/shapes that are not represented in the PCA plot. The same in Fig 3H, where in the PCA plot there are pink triangles that do not match with the code legends.*

R10: We apologize for the confusion with the legends of Figures 2C and 3H, that we have made unambiguous in the revised version (as well as Figures S2B,C and S3C).

C11: *8. In the figure legends of Figures 2D, 2E, 2F, and 2H, the notation should be corrected from "OgtT931A/Y" to "OgtT931del/Y".*

R11: This has been corrected; many thanks for bringing it to our attention.

Significance

C12: *To investigate the function of OGT at specific developmental stages, the authors perturbed OGT's function in vivo by creating a murine allelic series featuring four single amino acid substitutions that variably reduced OGT's catalytic activity. The goal was to identify the direct effect of O-GlcNAcylation, using a sophisticated collection of genetic mutants to evaluate in vivo the role of this modification at early stages of development. Overall, the severity of embryonic lethality correlated with the extent of catalytic impairment of OGT, demonstrating that the O-GlcNAc modification is essential for early development.*

R12: We thank the reviewer for their clear understanding of our work and their appreciation of the biological importance of the findings.

Reviewer #3

Full Revision

Evidence, reproducibility and clarity

C13: *This is a conceptually interesting paper that attempts to leverage the knowledge of OGT catalysis to begin to dissect OGT function. The evidence is presented I a straightforward fashion and is in general well documented. The breeding strategies are well informed and the paper draws heavily on previous work carried out in the mouse.*

R13: We greatly appreciate the overall supporting review. However, we fail to understand what they mean with "*the paper draws heavily on previous work carried out in the mouse*". This comment may stem from a misunderstanding because this work is not based on any previously published study. Specifically, neither the seven murine alleles presented and analyzed nor the single embryo-transcriptomic data sets on which our conclusions are based have been published elsewhere.

To put this work into context, before our study there were two seminal studies published two decades ago that reported the essential role of *Ogt* for mouse development, but no molecular profiling was performed (10.1073/pnas.100471497, 10.1128/mcb.24.4.1680-1690.2004). The two *Ogt* loss-of-function alleles studied in these papers were deemed as not suitable for interrogating molecular phenotypes because they caused cell death that confounds molecular profiling and embryonic lethality at implantation, thus preventing study of the sexually-dimorphic role of *Ogt* placenta. To overcome this long-standing problem, we created new seven murine alleles, which allowed us to tease apart molecular phenotypes at key stages of mouse embryonic development, focusing on the blastocyst and the placenta.

Significance

C14: *The paper describes tools which will help dissect the many potential roles of O-GlcNAc addition in early development. As it stands, this is a descriptive manuscript that will lead to hypothesis generation and testing and this should not be undervalued. The biological reagents produced and characterized will be of general interest to the field. Most of the findings presented represented a verification of existing ideas in the field but this is not meant as a criticism since part of the motivation for the approach was to generate a reproducible system for analyzing the biological phenomena.*

R14: We thank the reviewer for their appreciation of the importance of experimentally testing ideas shared in the field without direct evidence.

However, we must respectfully disagree with the qualification of "*descriptive manuscript*". This qualification may stem from the particularly difficult challenge to accessing the molecular details on how the O-GlcNAc modification exerts the biological functions we report. We are fully cognizant of the limitations of the study that we discussed in the discussion section and in R20.2. However, we feel that the adjective "*descriptive*" is not a fair qualification because we provide numerous novel functional evidence. Specifically, we introduce two novel orthogonal *in vivo* perturbations for endogenous *Ogt* that allowed us to interrogate for the first time its function in the developing mouse embryo. These perturbations allow us to draw causative conclusions (not descriptive) on the essential role of the O-GlcNAc modification itself for preimplantation development, its sexually-dimorphic role in the placenta and its requirement *in vivo* for the stable repression of retrotransposons.

C15: *There are perhaps some bioinformatic shortcuts taken that may need to be corrected upon thorough review. These do not lessen the overall impact of the contribution.*

R15: All the code written for the bioinformatic analyses performed in this study is publicly available: https://github.com/boulardlab/Ogt_mouse_models_Formichetti2024. The reviewer needs to specify which bioinformatic analysis they suggest could be improved.

Reviewer #4 (Evidence, reproducibility and clarity (Required)):

Summary

C16: *O-GlcNAcylation is the fundamental post-translational modification of numerous nuclear and cytosolic proteins. OGT is the sole enzyme catalyzing O-GlcNAc addition onto the proteins. The essentiality of OGT for early development and cellular viability has been established by using OGT-KO mice and cell lines. However, it remains to be elucidated whether the catalytic activity of OGT is required for the early development, and if the catalytic activity of OGT is required what are the functions of OGT or O-GlcNAcylation in early development due to a lack of appropriate mouse models. To overcome the technical difficulty of manipulating the levels of O-GlcNAcylation in early embryos, Formichetti et al. created the series of four mouse models (OgtY851A, OgtT931A, OgtQ849N, and OgtH568A) with different OGT activity by introducing single amino acid substitution in the catalytic domain. By analyzing the inheritance of the hypomorphic OGT alleles and the lethality of mouse embryos, they discovered OGT activity is a critical factor for early development. Subsequently, RNA-seq analyses with two mouse models showing the maternal inheritance of the hypomorphic OGT alleles indicated that sever hypo-OGT activity altered transcription and silencing of retrotransposon in preimplantation development while mild reduction of OGT's activity affected placental development in a sexually dimorphic manner rather than preimplantation development. Furthermore, to study the function of OGT at specific developmental stages, they developed a mouse model bearing endogenously AID-tagged OGT for acute degradation of OGT. Although the degron system wasn't efficient in preimplantation embryos, they discovered quick transcriptional changes upon OGT deletion in MEFs. The quality of the manuscript is good because the question to be solved was appropriately set, the approach was well designed, and their findings were interesting, although their writing was sometimes hard to understand as I raised in my following comments. Nevertheless, there are several points to be fixed before being published.*

R16: We thank the reviewer for their clear understanding of our work and their appreciation of the biological importance of the findings. Your comprehensive review of the manuscript and the questions you raised were extremely helpful in improving the manuscript and fully addressing its limitations. Below, we respond to comments in full, have revised the manuscript to improve clarity and have included novel results.

Major Comments

C17: *1. Although the authors showed in vitro activity of each mutant of OGT used in this manuscript by referencing the previous literature, they never showed the levels of global O-GlcNAcylation (and*

OGT itself) in their established mouse embryos. Although it could be impossible to determine O-GlcNAc levels in OgtQ849N and OgtH568A embryos because of the lack of germline transmission and founder line, respectively, they could do that in OgtY851A and OgtT931A embryos. Given that Y851A and T931A mutants had similar VMAX/KM with different VMAX, it is possible that their activity is comparable or Y851A has even lower activity in vivo depending on the concentration of UDP-GlcNAc in embryos. Therefore, it is critical to assess whether in vivo OGT activity is correlated with that in vitro as expected to conclude that severity of sub-Mendelian inheritance is proportional to the reduction of activity of OGT in vivo. Moreover, since the authors developed the elegant system to deplete OGT, the activity of Q849N and H568A mutant OGT can be examined at least in cells by expressing them in MEFs with OGT-degron system. Thus, I propose determination of global O-GlcNAc levels compensated by OGT levels by western blotting in OgtY851A, and OgtT931A embryos or MEFs with the OGT degron system re-expressing the individual four mutant OGTs. If the protein amount is insufficient for western blotting in the embryos because of the sizes of the earlier stages of embryos, I believe the author could address this by utilizing immunofluorescence as shown in Figure S5.

R17: We fully agree that this is an important point that requires revision. The only mutation for which the level of O-GlcNAc and OGT can be assessed by western blot *in vivo* is Y851A, the other mutations resulting in embryonic lethality before the blastocyst stage.

We have included in the revised manuscript western blot analyses of protein expression for OGT, OGA and O-GlcNAc levels in the placenta of the *OgtY851A* mutants (new Figures 3C,D). The new data show that OGT is upregulated at the protein level in homozygous females, in good agreement with our transcriptomic analysis. Furthermore, O-GlcNAc levels were slightly reduced in homozygous and hemizygous placentae thus showing the impact of the point mutation on global O-GlcNAc levels in the placentae. Moreover, the analysis of OGA protein level unexpectedly revealed the enrichment of a previously uncharacterized OGA fast migrating isoform in hemizygous and homozygous placentae.

We agree that it would be informative to compare O-GlcNAc levels in *OgtT931A* versus *OgtY851A* embryos. A comparison implies performing the experiment at the same developmental stage, which has to be the blastocyst stage or prior because *T931A/Y* embryos die around implantation. The blastocyst being made of approximately 140 cells, it would require to pool many single blastocysts to obtain the necessary protein input for western blot. We are not aware of another study performing western blot with pooled blastocysts. An additional great challenge for this experiment is the necessity to genotype and sex the blastocysts before pooling. Thus, the feasibility of this experiment is uncertain.

As an alternative, the reviewer suggests measuring O-GlcNAc levels in the degron MEFs after introduction of OGT transgenes bearing the mutation studied. This experiment would not be conclusive because of residual O-GlcNAc after OGT degradation (Figure S4E). Furthermore, the O-GlcNAc proteome is dynamic during development (as shown in the developing brain by Liu *et al.* https://doi.org/10.1371/journal.pone.0043724), therefore the MEFs results would have limited value to explain our results in the early embryo.

In sum, available technologies to quantify O-GlcNAc (e.g. western bot, mass spectrometry) are inadequate for low input samples as the early embryo. However, our series of hypomorphic alleles backed up with *in vitro* enzymology measurements brings indirect evidence to this question.

Specifically, the qualitative correlation between the measured OGT activity *in vitro* and the developmental phenotype indicates that the resulting relative levels of O-GlcNAc are consistent with *in vitro* measurements.

C18.1 : *2. I didn't understand why the authors couldn't find any founder lines of the OgtH568A mutant. Was that because mosaic mice with OgtH568A mutation are lethal?*

R18.1: To answer to this question, it is important to recall two key features of the biological system:

1) The mutation *H568A* was reported to disrupt the glycosyltransferase activity completely (10.1038/nsmb.1443). Hence, OGT-H588A is catalytic dead.

2) We performed the CRISPR-HDR targeting in the 1-cell embryo.

Based on these premises, the absence of F_0 with the *OgtH568A* mutation (0/31) suggests that introducing this mutation causes embryonic lethality in both males and females. This hypothesis is consistent with the previously reported lethality around implementation of *Ogt-null* alleles (10.1128/mcb.24.4.1680-1690.2004). It is possible that the sgRNA is very efficient and results in homozygous mutations in all female zygotes injected (as we have not obtained heterozygous females bearing these mutations). High efficiency of the targeted mutagenesis in the zygote results in mutants where all or the majority of cells bear the mutation (no or low mosaicism). The high number of microinjections performed (416 embryos over the 3 injection sessions) allows us to make these claims.

C18.2 : *Also, I believe there was no explanation why the OgtQ849N allele showed no maternal inheritance. Was that because Q849N possesses enough activity for sustaining mosaic embryos, but not oocytes? The authors should better explain these points in the manuscript text.*

R18.2: Thanks for this comment, we agree that this maternal effect phenotype demands further explanation.

The phenotype observed suggests two possibilities: either that the oocyte cannot maturate or that the cleavage-stage embryo cannot develop with the resulting lower levels of O-GlcNAc. The cleavage-stage embryo does not transcribe a catalytically active OGT before the 8-cell stage and thus relies on the OGT protein inherited from the oocyte until this stage (https://doi.org/10.1101/2024.01.22.576677).

Thank you for this comment, we added this interpretation of the result in the text: "*The lack of maternal transmission of the Q849N allele from seemingly mosaic founder females is likely explained by the reliance of the cleavage stage embryo onto the oocyte payload of OGT and O-GlcNAc modified proteins. Specifically, Ogt's exons encoding for the catalytic domains are not detectable before the 8-cell stage, while OGT full-length protein is present and thus maternally inherited (Formichetti et al, 2024).*"

C19: *3. The authors serendipitously found a T931del-allele in the "WT" allele of the OgtT931A line, and suggested that T931del had milder activity loss, although the lethality of embryos was greatly*

Review COMMONS

mitigated. Nevertheless, transcriptome analyses in male blastocysts revealed that 120 genes' expression was changed in T931del/Y males. This raised the question about which mutant OGT has higher activity, Y851A or T931del. I think comparing the activity of Y851A and T931del mutants in MEFs with OGT-degron system is important to confirm the proportional relationship between activity and phenotypic severity.

R19: We agree that it is a limitation that the effect of the *T931del* mutation on OGT activity has not been biochemically characterized. However, the important point here is that our assessment of phenotypic severity based on maternal inheritance of the mutant allele and embryonic lethality is based on the point mutations for which the catalytic activity has been determined, namely Y851A, T931A, Q849N and H568A, but not T931del.

We studied the serendipitously discovered *T931del* mutation to obtain transcriptional insights in the blastocyst. Because the deleted residue T931 is key for the binding to the donor substrate, we can reasonably assume that this mutation affects the catalytic activity, albeit to an undetermined level.

Hence, our conclusions regarding the requirement of O-GlcNAcylation for development are unaffected by the lack of biochemical knowledge on T931del.

C20.1: *4. Regarding transcriptomes of T931del/Y, the authors found the upregulation of proteasomal activity and stress granules along with the downregulation of amino acid metabolism, mitochondrial respiration, and so on. To validate the results, the authors should perform qPCR on several up- or down-regulated genes.*

R20.1 : We agree that, in principle, qPCR validation is suitable. However, this validation experiment is particularly expensive in this case because of the requirement of numerous CRISPR zygote pronuclear injection sessions.

The conclusions of the RNA-seq analysis are strongly supported by a high number of biological replicates (n=10). This high number of biological replicates was essential to obtain sufficient statistical power to quantify with a high level of confidence transcriptional changes of low magnitudes (below 2-fold change, see R5.1 and R5.2).

Therefore, the qPCR validation experiment would require to repeat the CRISPR zygote pronuclear injection sessions with the same high number of animals. This represents a major investment in experimental work and the sacrificing of about 40 animals. Importantly, the RNA-seq results presented are authoritative because of a high number of biological replicates and high number of sequencing reads per sample. Thus, we argue that qPCR validation is not essential and thus the high cost of this experiment is difficult to justify.

C20.2: *In addition, according to Figure S2E, the authors pointed out that at least for genes upregulated in OgtT931A embryos, the changes were not explained by a developmentally delayed transcriptome, suggesting that upregulation of these genes was the cause of developmental delay. Therefore, I strongly*

encourage them to discuss in the manuscript text how up-regulated genes could contribute to developmental delay.

R20.2: Throughout the manuscript, we have been cautious to avoid establishing causal relationships between the differentially expressed genes uncovered and the developmental phenotypes (e.g. delayed development). There are two main obstacles which we believe prevent us from establishing causality with the data available. Firstly, it is not possible to disentangle differentially expressed genes and developmental delay (in other words, we have no way to tell which is the cause and which is the consequence). Secondly, O-GlcNAc modifies over 5000 proteins and the developing embryo is a particularly dynamic system; thus we cannot know whether the differentially expressed promoters are direct targets of O-GlcNAc modified proteins (or alternatively secondary effect of another molecular alteration, for example of the proteome). We discuss this limitation of the study in the discussion section.

C21: *5. Regarding the transcriptome in OgtY851A mice, Y851A/Y male mice had huge transcriptomic differences, while Y851A/Y851A female mice barely had any. Although it seems to agree with the number of Ogt alleles, I wonder whether other X-linked genes expressed higher in female placenta as shown in Figure 3C could attenuate the effects of decreased OGT activity. I don't think this possibility can be excluded, unless the authors further decrease OGT activity in Y851A/Y851A female placenta and obtain the similar results as for male placenta. Or if they compared the levels of global O-GlcNAcylation between Y851A/Y and Y851A/Y851A mouse placentas and discovered they had similar levels of O-GlcNAcylation, then the authors could conclude that the number of Ogt alleles was not the reason of sexual-dimorphism. The authors should determine the levels of O-GlcNAcylation in Y851A/Y and Y851A/Y851A mouse placentas and/or at least discuss the above possibilities in the manuscript text.*

R21: Thank you for the thoughtful feedback. We agree that the most likely explanation for the higher sensitivity of males placenta as compared to females to OGT reduced activity is the difference in *Ogt* copy number, especially because *Ogt* escapes X-chromosome inactivation in the placenta (new Figure S3A).

Western blot quantification of global O-GlcNAc levels was now performed (new Figures 3C,D). We measured similar level of O-GlcNAc in *Y851A/Y* and *Y851A/Y851A* placentas (lowered than WT males in both cases), but we cannot exclude that the WB does not have the dynamic range required to detect a subtle difference. In fact, female homozygous were expected to have an intermediate level between WT males and hemizygous males, and the difference between the two male genotypes (also considering sample-to-sample variability) is already small when quantified from the blot (new Figure 3D). It is possible that a X-linked modifier attenuates the impact of hypo-O_GlcNAcylation in female mutant placenta in the case of identical O-GlcNAc levels in homozygous females and hemizygous males. Thank you for the idea that we included in the revised manuscript:

"*Of note, the lower sensitivity of the homozygous females' transcriptome to Ogt disruption (Fig. 3F,I and S3B) seems difficult to reconcile with their lower O-GlcNAc level comparable (lower) O-GlcNAc*

level to the hemizygous males (Fig. 3C). It is possible that the western blot technique is not sensitive enough to detect subtle differences in O-GlcNAcylation. An alternative hypothesis, if O-GlcNAc levels were truly identical between Y851A/Y and Y851A/Y851A, could be the existence of a modifier in female that could be a XCI-escapee."

C22: *6. In terms of the transcriptome in OgtY851A mice, similar to comment 4, the authors should confirm their transcriptomics data shown as Figure 3D by qPCR. In addition, the authors should describe the potential mechanisms by which the differentiation of precursor cells of LaTPs and JZPs were disrupted. Were master regulators of the differentiation known to be O-GlcNAcylated and loss of O-GlcNAcylation perturbed the function?*

R22: As for the whole embryo discussed in R20.2, we also interpret cautiously the gene expression phenotype observed in the placenta. Specifically, we state in the manuscript that it could either be caused by an impact of lower O-GlcNAcylation on placental differentiation or by a general delay in placentation or in the development of the embryo as a whole. The hypothesis of a general delay (of the whole embryo and/or of placental formation specifically) is supported by the downregulation of essentially all markers of more differentiated cell types and the upregulation of the precursor marker. We favor this hypothesis because it is consistent with what observed with the *T931* mutants and also with the enzymatic removal of O-GlcNAc in the zygote (Formichetti et al., 2024 BioRxiv). Because of the thousands of O-GlcNAcylated proteins present in the cell, it is impossible to know which is the responsible molecular mechanism, which could even start at much earlier stages.

Minor Comments

C23: *1. Regarding DFP461-463 mutant, I couldn't understand the point of this figure because the results had no difference, and the meaning of the mutation was quite different from the others. Thus, the figure was awkward and a little confusing to me. If the authors still want to include the figures, I would suggest that they should reorganize the position of the figure (maybe after figure 3 is better to show you had tried to investigate the effects of nuclear localization of OGT on the changes of transcriptomes) and add some results. Since WT OGT seems to be localized mainly in the cytosol at steady state (Figure S1B and S1C), the effect of mutation on its nuclear localization should not be obvious. Therefore, it is difficult to conclude the mutation had no effect on the nuclear localization unless the ratio of nuclear and cytosol localization is quantified. Also, I wonder whether the O-GlcNAc levels of nuclear and cytosolic proteins in the mutant cells were comparable to those in WT cells. If this is the case, the results would also support the authors' conclusion.*

R23: We took the comments on board and made it clearer that the rationale for the *DFP461-463* mutant was an attempt to separate OGT's nuclear and cytosolic functions. We fully agree that these results are peripheral, and thus we presented these results in Supplementary Figure 1 (not in the main figure).

The biochemical evidence presented in Fig S1C shows that the genetic substitution of DFP to AAA on endogenous OGT has no detectable impact on its nuclear localization in primary MEFs. This result is far more authoritative than the evidence provided by Seo *et al.* 2016 (doi: 10.1038/srep34614), which

is based on the overexpression of OGT transgenes in HeLa cells. Importantly, Seo *et al.* 2016 did not assess the impact of their mutations on endogenous OGT.

We believe that the negative results we obtained with the *DFP461-463* mouse model shall be extremely valuable for the field. Firstly, science can move forward only if both negative and positive results are shared. In this specific case, we found that mutation of endogenous OGT in MEFs yielded to a different result than previously reported overexpression of the same mutant construct in HeLa cells. Secondly, we want to make the *Ogt-NLS-* mouse model available for further investigations.

C24: *2. Since OGT or O-GlcNAcylation regulates chromatin status, the authors analyzed the gene expression profiles of retrotransposons in T931del/Y or T931A/Y mice. Is it possible to investigate if the release of gene silencing is also seen in non-retrotransposon genes? I assumed retrotransposons might be a well-established system to analyze gene silencing status, however, if the authors could find similar effects on genes other than retrotransposons, that would be highly valuable.*

R24: This is an interesting idea. This notion refers to the activation of promoters that are normally epigenetically repressed (e.g. silent despite the presence of all trans-active factors required for their expression). Epigenetically repressed promoters include retrotransposons, imprinted genes and germline specific genes that are normally expressed in germ cells and maintained in a repressed state in somatic cells (10.1038/s41580-019-0159-6). Testing of mono-allelic expression of imprinted genes required F1-hybrid. Thus, we assessed whether well-studied germline specific genes could be realized from silencing in *T931del/Y or T931A/Y* blastocyst and found no evidence for it (see dot plot below). The unbiased transcriptomic analysis presented in the manuscript shows that the product of upregulated genes are enriched in mRNA processing (Figure 2E), but these genes are not normally epigenetically repressed. Thus, contrary to retrotransposons, the role of O-GlcNAc at cellular gene promoters appears not to be linked to epigenetic silencing. This could be explained by the many different protein substrates for O-GlcNAc.

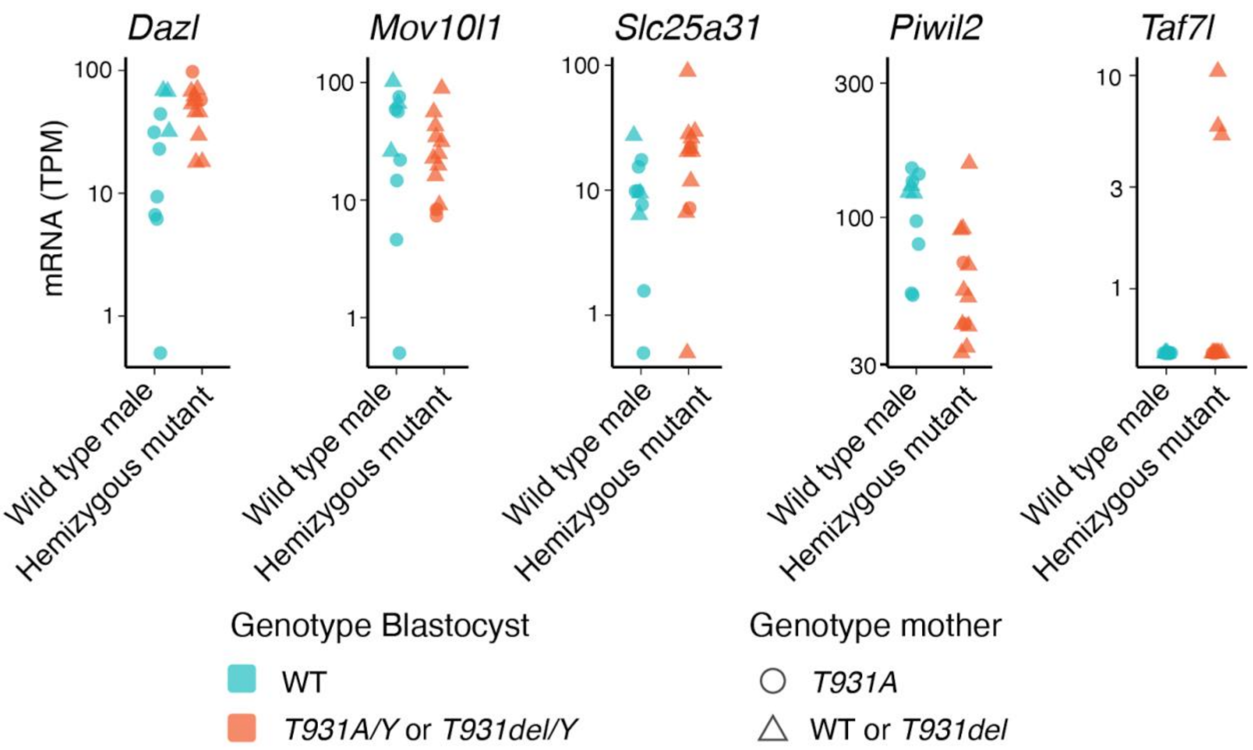

C25: *3. OgtY851A mice with milder OGT activity loss didn't exhibit impaired preimplantation development, but did display postimplantation development such as placental development, suggesting that O-GlcNAcylation of proteins required for preimplantation and postimplantation development relies on different degrees of OGT activity. I wonder whether global O-GlcNAc levels in embryos in preimplantation and postimplantation developmental stages are different or not. This might include both the pattern of blotting and intensities. The results would give the authors an explanation why the dependency on OGT activity was different in two developmental stages. Can the authors provide data? If not, then the authors should at least describe hypotheses in the manuscript to address these questions.*

R25: We recently reported that the subcellular patterns of O-GlcNAc are highly dynamic during preimplantation development (Formichetti *et al.* 2024, BioRxiv). The most striking O-GlcNAc remodeling we observed is the enrichment of nuclear O-GlcNAc as compared to cytoplasmic O-GlcNAc that is concomitant to embryonic genome activation (Formichetti *et al.* 2024, BioRxiv). We quantified the ratio of the nuclear/cytoplasmic signal by immunofluorescence, but absolute quantification is not possible with this method. Due to the limited number of cells of the preimplantation embryo, this analysis cannot be performed by western blot. Hence, there is no appropriate method to quantitatively compare O-GlcNAc levels between preimplantation and postimplantation embryos.

C26: *4. The authors' AID-degron system elegantly worked in MEFs but was inefficient in preimplantation embryos. I wonder if this was because of the high expression of the shorter isoform of*

OGT detected as OGTp78 in the author's western blot. Is it possible to examine this possibility in the embryos? Either way, the authors should describe a potential explanation for why the efficiency in the embryos was low. In addition, the authors should describe why they inserted the AID tag only into the longest OGT isoform.

R26: This is a good point. The smallest isoform OGTp78 bears the catalytic domain and thus can partially compensate for the degradation of OGTp110. Note that the level of OGTp78 is low and does not increase upon OGTp110 degradation; thus a compensation can only be partial (Figures S4A and S4D). Alternative hypotheses for the ineffectiveness of the degron system in *ex vivo* grown embryos include: i) the expression level of OsTIR that may be too low in the early embryo (Rosa26 promoter not being activated at EGA), ii) a possible steric hindrance of the N-ter AID tag in these cells, iii) the lower concentration of Auxin imposed by toxicity on the embryo is likely suboptimal. Testing these possibilities is very difficult in preimplantation embryos.

It is unclear how the OGTp78 isoform is produced; it was hypothesized to originate from an alternative transcription start site (https://doi.org/10.1007/s00335-001-2108-9). We initially attempted to target both isoforms by inserting the AID tag at the C-terminus, but we were unsuccessful in producing this mouse model. It is possible that the C-terminus that is near the catalytic site cannot tolerate the AID knock-in.

C27: *5. In Figure S1C, is the band detected right below OGTp78 in nuclei fractions non-specific or do both bands correspond to OGTp78 ?*

R27: To answer this question, a knockout control would be needed. OGTp78 being not targeted by our AID-degron, we cannot test the specificity of these bands using our perturbation tool kit.

C28: *6. Figure 1D top row third column: hemizgous -> hemizygous*

R28: Many thanks; the embarrassing typo has been corrected.

C29: *7. Figure 1D second row third column: hemyzygous -> hemizygous*

R29: Thanks for bringing this other typo to our attention, it is now corrected.

Reviewer #4 (Significance (Required)):

General assessment: strengths and limitations

C30: *Strength: This manuscript elegantly revealed the requirement of OGT in mammalian development by taking advantage knock-in mouse models with different OGT activity. In addition, the manuscript*

provided the interesting and important transcriptomics data in both pre- and post-implantation embryos of OGT mutant mice. These data sets could explain detailed mechanisms how OGT or O-GlcNAcylation regulates mammalian development in the future. Furthermore, development of AID-tagged OGT system would be a useful tool for other researchers studying OGT function.

Limitation: Although they found interesting changes in terms transcriptomes in developing mice with different OGT activity, they lack the data showing how these changes caused the observed phenotypes. In other words, there are less mechanistic insights behind the developmental problems seen in mice with different OGT activity.

In addition, although I agree the question about whether OGT activity itself is crucial for the early development of mammals has not been completely solved for a long time, I assume people thought OGT activity is actually important for the mammalian development thorough the observation of OGT-linked congenital disorders of glycosylation.

Therefore, I would say the novelty of the manuscript is a little less impactful. Furthermore, although AID-tagged OGT system revealed fundamental questions regarding the transcriptional changes upon acute depletion of OGT in cellular levels, the system was inefficient in mouse embryos. So, they showed nothing about developmental-stage specific requirements of OGT.

Advance: The manuscript can fill a current gap regarding requirement of OGT in mammalian development. Also, the manuscript developed a series of mutant mice with different OGT activity and an AID-tagged OGT mouse line. These mice provide technical advances.

Audience: The manuscript will be interested in researchers in specific fields such as glycobiology, developmental biology, and clinical fields.

Describe your expertise: Biochemistry, Glycobiology, Cell biology

R30: We are thankful for the constructive and supportive review.

We fully agree with the limitations of the study and discussed them in the manuscript. Our *in vivo* approach revealed the most phenotypically relevant transcriptional phenotypes resulting from OGT catalytic impairment during embryonic development. We make the mouse models created for this study available to the community to facilitate follow-up studies aiming at exploring the underlying molecular details.

As pointed out in the comments, the requirement of OGT glycosyltransferase activity for mammalian development was widely assumed by the field, but this belief was without direct experimental evidence. This study provides the first *in vivo* evidence for this important conclusion.

Conclusion: The reviewers' comments were tremendously useful to improving the clarity of the manuscript and adding important new *in vivo* evidence. We note that none of the reviewers provided any reason to doubt our important conclusions:

- The demonstration that the enzymatic activity of *Ogt*, thus the O-GlcNAc modification itself, is essential for preimplantation development.
- The finding that a mild reduction of OGT's activity is sufficient to perturb the silencing of multiple families of retrotransposons in the growing embryo.
- The indication, from transcriptomes of hypo-O-GlcNAcylated embryos, of a developmental retardation upon a mild O-GlcNAc perturbation.
- The discovery that OGT's rapid depletion *in vitro* downregulates basal cellular function, including translation. This result provides mechanistic support to the embryonic growth delay resulting from decreasing O-GlcNAc *in vivo*.
