# Supplementary material for: Genetic gradual reduction of OGT activity unveils the essential role of O-GlcNAc in the mouse embryo
Source: PLoS Genet. 2025 Jan 9;21(1):e1011507. doi: 10.1371/journal.pgen.1011507 (PMC11717234; doi:10.1371/journal.pgen.1011507)
Supplement: Attachment — Submitted filename: Response_to_reviewers_Formichetti.pdf [file pgen.1011507.s014.pdf]

Responses to Reviewers

We thank the three reviewers for their interest in this manuscript, their positive and constructive feedback. Please find below a point-by-point response, which includes summaries of changes made in response to these comments. The original reviewer comments are replicated in full in black text, while our responses are in blue text.

<u>Reviewer #1</u>: In this manuscript, the authors demonstrated that catalytic activity of OGT is crucial for embryonic development by using mice having single amino acid mutations on its catalytic domain. The mutant mice showed altered gene expression including upregulation of some retrotransposons, which may explain developmental delay and sub lethality of the mutant embryos.

The experiments are conducted well, but the manuscript is long and often complicated. I would recommend that the authors edit the manuscript in more concise style. Descriptions are often confusing as listed below, which should be edited. In vivo degron experiments are not informative and should be deleted from this manuscript.

We agree in full, we have edited the text to improve clarity and conciseness. The *in vivo* degron experiments are now provided as supplemental information.

Specific comments:

1. Page 3, 1st paragraph; 'The lack of maternal transmission of the OgtQ849N allele from seemingly mosaic founder females is likely explained by the reliance of the cleavage stage embryo on the oocyte payload of OGT and O-GlcNAc modified proteins.'

It is not clear for me what this sentence means, because mosaic founders seem to give rise to wild type embryos as well.

The reviewer is correct: the heterozygous F0 *OgtQ849N* female transmitted the *Ogt-WT* allele but never the *Ogt-Q849N* allele.

The transmission of a mutation to the next generation implies that competent germ cells carry this mutation. There are two possibilities to explain the lack of transmission from the F_0 generation (CRISPR modified animals) to the F_1 generation:

1. The germline does not bear the mutation. This possibility is unlikely because the zygote was targeted with Cas9, four F_0 founders were tested while the F_0 females *Ogt-Y851A* produced in parallel transmitted the mutation at the expected frequency.
2. The mutated germ cells do not produce viable embryos.

Considering that i) neither F_1 male (1 *Ogt* allele) nor heterozygous females (2 *Ogt* alleles) were recovered, and ii) the mosaic *OgtQ849N* heterozygous females founder were viable, it is reasonable to consider as the most likely explanation for the absence of F_1 heterozygous *Ogt849N* that the embryos with at least one strong hypomorphic *Ogt* allele die during early development. It cannot be excluded that this very disruptive mutation could be incompatible with the viability or the maturation of the oocytes or male germ cells. We included this possibility in the revised manuscript.

2. Page 6, 2nd paragraph; 'especially considering that a posteriori sequencing-based genotyping of the pair of control mothers revealed that only one of them was OgtT931del/+ while the other WT.'
It is not clear for me what this sentence means. Why control (wild type?) mothers give rise to OgtT931del/+?

The explanation is in the sentence that precedes the quoted text:
"*We manually inspected the RNA-Seq reads mapping to Ogt and unexpectedly uncovered the presence of an additional mutation of the T931 codon in the blastocyst population produced by the control females, namely a precise deletion of T931 (Fig. S2A).*"

In other words, we discovered *a posteriori* that one of the control mothers had a deletion of the T931A codon (we named this mutation *Ogt*-T931del). This happened because the control mothers were littermates of the F_0 *Ogt*T931A and were genotyped by PCR as wild type. It turned out that Cas9 with the injected sgRNA caused the deletion of 3 nucleotides. We took advantage of this mutant to investigate the molecular phenotype of blastocysts bearing the precise deletion of an essential amino-acid (T931) for the binding of the donor substrate within the catalytic core.

3. Page 8, 3rd paragraph, Fig. 3D; 'the total O-GlcNAc level was reduced in hemizygous males and homozygous females (Fig. 3C,D)' Is it statistically significant?

We did not include the p-value for this figure because the statistical power given by two biological replicates is too low. We provide in the figure below the p-values for the reviewers (see page 3).

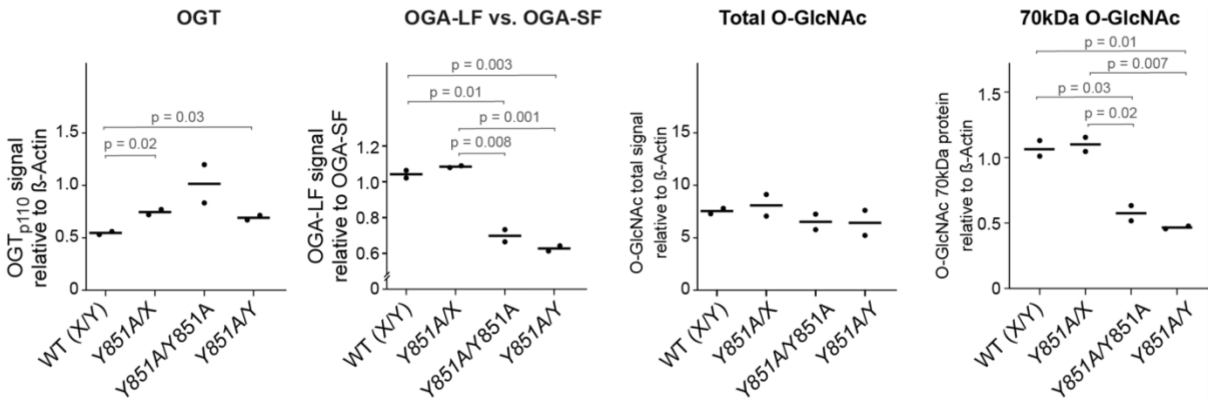

Significant p-values (p<0.05, two-tailed t-test) are shown, the comparisons with non-significant p-values are not shown. For the total level of O-GlcNAc, the comparison is not significant. However, it is noteworthy that O-GlcNAc levels significantly decrease when considering the most abundant ~70kDa band. In the manuscript, we present the raw WB results and unbiased quantification of O-GlcNAc using the signal from the whole lane.

4. Page 9, 1st paragraph; 'Markers for precursor cell types of both labyrinth and junctional zone (LaTPs and JZPs) were all upregulated in OgtY851A/Y placentae, while markers for matured cell types (SynTs, sTGCs, SpT), which increase in percentage from E10.5 to E12.5 [69], were all downregulated (Fig. 3I). Of note, downregulation was also observed for markers of endothelial cells, which also compose the fetal-maternal exchange barrier but are not of trophoblast origins (Fig. 3I).'
This sentence is unclear in terms of correlation to Fig.3I. Referring Fig.3H may be more suitable.

We thank the reviewer for their comments. This was a mistake from us for which we apologize: the text was referring to Fig. 3H of the submitted version of the Figures, we corrected this in the revised manuscript by inverting Fig.H and I in the revised figure, i.e. the gene set enrichment analysis with placental clusters will now be panel I and the one with GO terms panel H, since GO terms are mentioned before placental markers' enrichment in the text.

5. Page 10-11, Fig.S5; Because AID-OGT experiments in vivo are not informative due to inefficient AID degron, I recommend to remove this part.

We agree and moved these results in Supplementary text. While the *in vivo* degron experiments were not informative on the function of O-GlcNAc in the preimplantation embryo, they provided new information on the limits of the in vivo AID-system, which should be of interest for the field of mouse development.

6. Page 13, 5th paragraph; 'The developmental requirement of OGlcNAcylation was not obvious'
It is not clear for me what this sentence means.

We thank the reviewer for this comment that helped us to improve the clarity of the discussion. The revised text reads as follows:
"While it has been known for over two decades that maternal Ogt is essential for embryonic survival [15,16], it remained unclear whether non-catalytic roles of OGT could be the ones responsible for the lethal phenotype. In particular, OGT's N-terminus composed by 13 tetratricopeptide (TPR) repeats mediates interactions with various proteins [76] and could potentially also play an essential structural role."

Reviewer #2: The manuscript by Formichetti et al. addresses a pertinent issue regarding the developmental role of O-GlcNAc in mouse development. Building upon their prior experiments, the authors have performed an impressive amount of work to establish mouse models that allow for the dissection of the catalytic function of OGT rather than the whole complex. Importantly, the manuscript reports on mouse mutants showing a range of catalytic activities. Using these new tools, the authors first focus on the early developmental phenotypes of the severe hypomorph mutant. This analysis revealed that fewer blastocysts carrying the mutation are found in both males and females, which relates to significant dysregulation of several genes, including those linked to metabolism. Similarly, there is mild transposable element upregulation. Overall, mutant embryos show signs of developmental delay and metabolic dysregulation. In the case of a mild reduction in OGT activity, the authors report a reduced litter size and sex-specific placental phenotypes. Specifically, male placentas show significant dysregulation of multiple pathways and signs of delayed placental differentiation.
Overall, this is an incredible amount of mouse work that reports on interesting and novel OGT-associated phenotypes. I only have minor points of criticism that could be addressed before publication:

We thank the reviewer for their supporting statements. We are grateful that they appreciated the strength of our work and recognized its novelty.

1. Figure 2F/S2E reports some developmental delay; however, the analysis seems a bit cherry-picked. I would suggest performing GSEA where all genes are ranked based on their developmental dynamics between 8c and E3.5. Next, one can look at differentially expressed genes in mutants and see if they are enriched for such developmental genes.

We thank the reviewer for this excellent suggestion. We performed this analysis and included the result in Figure 2F. This new analysis provides additional support to our finding of a developmental delay of the *Ogt*-hypomorphic male blastocysts. For convenience, the new Figure 2F is reproduced below:

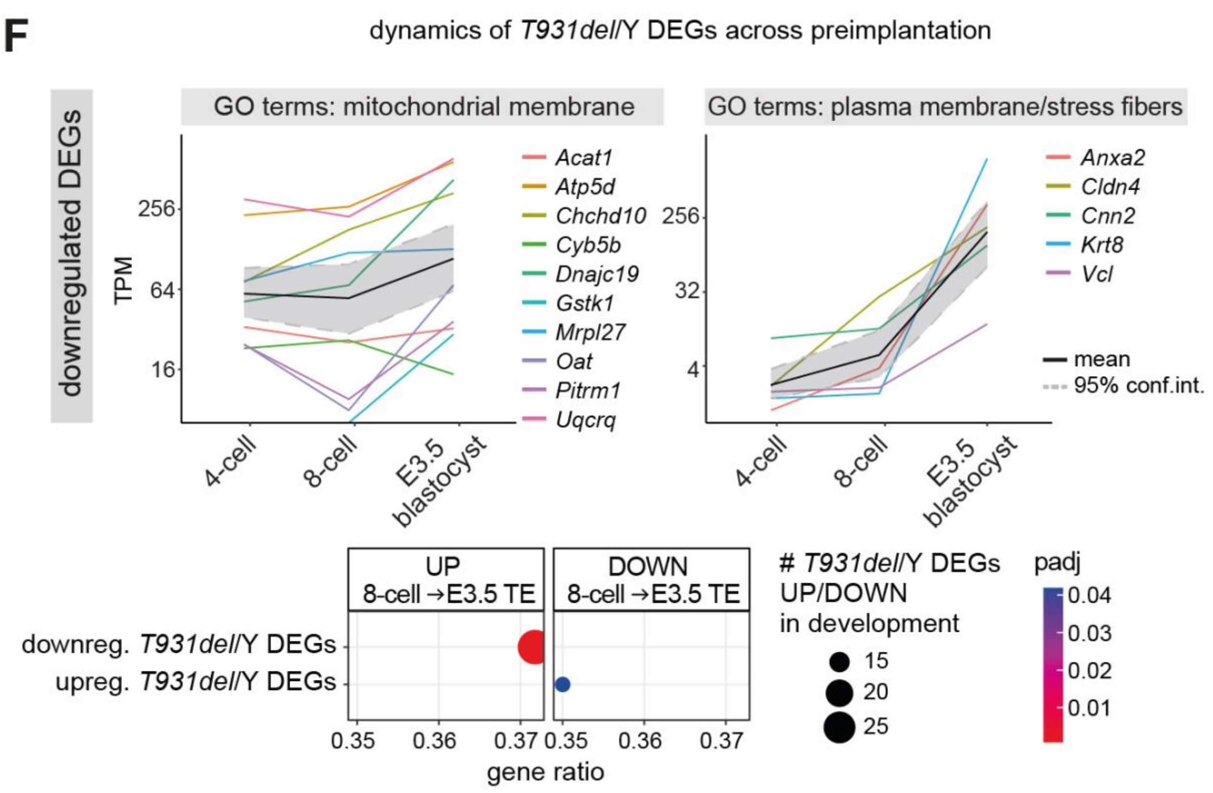

2. In general, the TE upregulation is mild, and there are also some downregulated TEs. Can we really say that there is mild upregulation? It's rather mild mis-regulation.

We observed that "*Some single repeats were downregulated, but they were generally lowly expressed, and their fold change was lower than upregulated repeats (Fig. 2H and Fig. S2G).*" While the upregulation of highly abundant TEs is robustly detected, we cannot exclude that the downregulation, even if significant, of lowly expressed TEs from single blastocyst RNA-seq data could be the result of fluctuating low-level noise (note the exponential scale in Fig 2H).

3. One limitation of the study is that the levels of O-GlcNAc are not always quantified. For T931A, there is no quantification (even by IF), while for Y851A, there is a nice WB, but I don't understand the quantification (Fig 3D). Why is the effect so mild in the quantification? The authors also state: "the lower sensitivity of homozygous females' transcriptome to Ogt disruption seems difficult to reconcile with their comparable (lower) O-GlcNAc levels to hemizygous males." However, I don't see these lower levels. What is this relating to?

We apologize for the lack of clarity on this point. To address this comment, we modify the quoted sentence by:
"*The lower sensitivity of the homozygous females' transcriptome to Ogt disruption (Fig. 3E,F and S3B) seems difficult to reconcile with their comparable O-GlcNAc level to hemizygous males, namely lower than in WT males and heterozygous female placentae (Fig. 3C).*"

We also now specify in the legend of Figure 3D that the O-GlcNAc quantification was performed by measuring the optical density of the entire western blot lane for each sample. In our response to question 3 by Reviewer 1 (page 2), we expend further on the WB quantification and provide the p-values (with the limitation that they are computed with only 2 biological replicates).

We propose that three factors could explain the very overall mild reduction of global O-GlcNAc levels in Y851A placentae: i) the upregulation of *Ogt* and downregulation of *Oga* can at least partially compensate *Ogt* hypofunction, ii) the limited sensitivity of antibody-based detection method to detect substoichiometric changes, iii) hypo-O-GlcNAcylation is not even but preferentially affect specific proteins (the most heavily glycosylated as shown in the result presented page 3).

4. Authors mention that T931del shows signs of translational stress, but I don't understand what this is based on.

The upregulation of proteasomal activity and stress granules (Fig. 2E) could be signs of a translational stress response. Moreover, this upregulation cannot be explained by a developmentally delayed transcriptome, which is instead the case for the downregulation of mitochondrial or cell adhesion-related GO terms (Fig. 2F).

5. Some explanation of what Fig 3G represents would be helpful.

We apologize for the insufficient explanations on Figure 3G. In response, we wrote a more detailed figure legend in the revised manuscript.

The plot in Figure 3G shows the functional-*Ogt* dosage-sensitive gene expression dysregulation: highest in hemizygous males, intermediate in homozygous females, which likely compensate thanks to the two copies of *Ogt*, and lowest in heterozygous females, which due to the mutation are comparable in terms of differential gene expression to WT males.

Stylistically, I would like to see this text shortened, and I also miss some conclusions after individual parts.

We agree with this point that was also raised by Reviewer 1. We have edited the manuscript to make it more concise and improve clarity, we hope, to the reviewers' satisfaction.

Nevertheless, this is an interesting study that seems suitable for PLOS Genetics.

We thank once again the reviewer for their insightful and supporting review and advocating for publication in PLoS Genetics.

Reviewer #3: Formichetti and colleagues put forward an interesting, thorough investigation of the importance of OGT in vivo. This is an effort marked by many challenges, some of which unsurmountable within the time frame and the current state of affairs of competitive fundamental research involving mouse models.
Due to OGT being essential for development, the author's efforts to characterize a series of alleles, some of which allowing development, have allowed unprecedented advances in characterizing the consequences of OGT defects in mutants. Incidentally, together with the difficulties of working in vivo, this makes the interpretation of data much more tricky as the effects are milder and the complexity is high. The authors however stuck to high level scientific rigor and did an amazing job making claims that are true to their data, being open about the shortcomings, and discussing the possibilities of what the data means in light of what is known. This work has also been seen and reviewed by other 4 reviewers, whose questions that where within reasonable scope were addressed and discussed carefully by the authors.
In light of all this, the opinion of this 5th reviewer is that this work has to be accepted and published. It is a large effort for a fairly small conceptual advance, but it is a real advance and

it does set a new high standard for future efforts aiming at characterizing the role of OGT in mammalian development.

I would like to congratulate the authors on the great work and sticking to a good scientific reporting of difficult findings.

We are genuinely thankful for the reviewer's enthusiasm and interest in our work. We particularly appreciate his recognition of the challenges we addressed in interpreting results obtained in perturbing *in vivo* the complex system that is the O-GlcNAc proteome. Finally, we are sincerely grateful for his thoughtful comments, appreciation of the scientific rigor of this work and advocacy for publication.

Have all data underlying the figures and results presented in the manuscript been provided?

Large-scale datasets should be made available via a public repository as described in the *PLOS Genetics* data availability policy, and numerical data that underlies graphs or summary statistics should be provided in spreadsheet form as supporting information.

Reviewer #1: Yes

Reviewer #2: Yes

Reviewer #3: Yes
